# Editorial: Changes in extracellular matrix associated with bone disorders

**DOI:** 10.3389/fendo.2024.1386459

**Published:** 2024-03-01

**Authors:** Caterina Licini, Xiao Lin

**Affiliations:** ^1^Department of Clinical and Molecular Sciences, Università Politecnica delle Marche, Ancona, Italy; ^2^Laboratory for Bone Metabolism, Key Lab for Space Bioscience and Biotechnology, School of Life Sciences, Northwestern Polytechnical University, Xi’an, Shaanxi, China; ^3^Research Center for Special Medicine and Health Systems Engineering, School of Life Sciences, Northwestern Polytechnical University, Xi’an, Shaanxi, China; ^4^Northwestern Polytechnical University-University of Alabama (NPU-UAB) Joint Laboratory for Bone Metabolism, School of Life Sciences, Northwestern Polytechnical University, Xi’an, Shaanxi, China; ^5^Research & Development Institute of Northwestern Polytechnical University in Shenzhen, Shenzhen, China

**Keywords:** extracellular matrix, bone disorders, biomarker, therapeutic drugs, osteoblasts, osteoclasts, irisin, mechanical properties

Extracellular matrix (ECM) plays an important role in the physiological maintenance of bone biology, and its alterations in structure and composition are involved in fragility fractures, bone deformities, and serious bone disability. The identification of novel biomarkers as well as the proposal of new treatments are useful to counter the effects of ECM alterations in bone diseases and the aim of this Research Topic has been to group some original articles and reviews within.

Zhang et al. investigated the role of chronic intermittent hypobaric hypoxia (CIHH) in osteoporosis (OP) triggered by spinal cord injury (SCI). In this work, the authors analyzed, in rats, parameters of bone mass and structure (i.e., Bone Mineral Density, and Histomorphometric parameters), markers of bone formation (procollagen type 1 N-terminal propeptide and osteocalcin in serum, ALP and OPG mRNAs in bone tissue), as well as markers of bone resorption (sclerostin in serum, RANKL and TRAP mRNAs in bone tissue). Their results showed that CIHH ameliorated effectively the OP condition in SCI rats compared with the sham groups by balancing osteoblast and osteoclast activities in rats, suggesting a treatment able to hamper the ECM alterations due to OP.

Yang et al. focused on the use of fluorescent oxidation products (FlOPs), already known as oxidative stress markers, as novel serum biomarkers of OP. They evaluated the serum levels of different FlOPs in OP subjects, forty-four cases and 88 matched controls (mean age: 68.2 years) were included. The results showed that higher FlOP_360 (excitation/emission wavelengths 360/420 nm) and FlOP_400 (excitation/emission wavelengths 400/475 nm) levels were associated with an increased risk of fracture, and this association was comparable for hip and non-hip fractures.

Long-term physical training affects the dynamic interaction of bone ECM with osteoblasts and osteoclasts and then influences the mechanical properties of bone tissue through changes in the composition of ECM. Song et al. presented a study to investigate the effects of blood flow restriction training (BFRT) on muscle strength, bone tissue structure material, and biomechanical properties in rats. They found that BFRT can improve the expression of bone turnover markers (PINP and BGP), which promote bone tissue formation. Moreover, BFRT could improve muscle strength and increase the positive development of bone turnover markers, thereby improving bone biomechanical properties such as bone elastic modulus and maximum load.

Irisin is a polypeptide consisting of 112 amino acids, which is cleaved and secreted by the fibronectin type III domain-containing protein 5 (FNDC5). 72% of circulating irisin is derived from skeletal muscle, and exercise will induce up-regulation of FNDC5. Liu et al. summarize the role of FNDC5/irisin in bone physiology and pathology. Recent studies showed that the muscle factor irisin is inextricably linked with bone health problems. Lower serum levels of irisin may increase the risk of bone fracture and lead to a series of bone diseases such as OP, osteoarthritis (OA), rheumatoid arthritis (RA), and osteosarcoma (OS). Therefore, irisin may be a predictor of bone diseases. In addition, irisin can be involved in regulating chondrocyte metabolism and reducing decreasing apoptosis of chondrocytes, with the regulation of ECMs (such as Collagen II, MMP-1, MMP-13, and ADAMTS-5), thereby slowing down the progression of OA. Studies have also found that irisin inhibits the decrease in bone mineral density and prevents bone loss in OP, has a therapeutic effect on RA, and suppressed the proliferation, migration, and invasion of OS. These results demonstrated the therapeutic effect of irisin on bone disease (Zhao et al.).

Osteogenesis imperfecta (OI) is a common systemic connective tissue disease caused by abnormal synthesis, exon coding, and synthesis of type I collagen, which is required for normal ECM function. A case report from Mai et al. was aimed to find out the genetic characteristics of a fetus with OI by whole exome sequencing (WES) of fetal aborted tissue and peripheral blood and to identify the cause of the disease. Interestingly, they found a novel de novo variant of COL1A1 (c.1309G>A, p. Gly437Ser), which is present in highly conserved glycine residues of Gly-X-Y sequence repeats of the triple helical region of the collagen type I α chain. Another case of OI was reported by Chen et al. They analyzed the whole blood samples of one OI patient and the normal control by RNA transcriptome sequencing. The results showed that 513 genes were differentially expressed in OI patients and the normal control. There was no expression of COL1A1 in OI patients, but the expression was present in normal control. The KEGG signaling pathways were mainly enriched in ECM–receptor interaction, including ITGA2B, LAMC1, LAMB2, COL6A2, ITGB3, THBS1, ITGB5, GP9, and ITGA1. This variation of type I collagen and the different expression of ECM recognition molecules, thus afflicting the molecular structure and signal transduction of bone ECM and triggering the OI disease.

All articles showed changes in ECM and the physiological role involved in different bone disorders, and therefore use a model system for distinct bone diseases, including osteoporosis, osteoarthritis, and spinal cord injury, to reveal ECM characteristics and changes in cells, tissues, and the entire organism. There are still many interesting facts to be explored, such as how ECMs interact with each other, how the mechanical properties of cells, tissues, and their microenvironment ECMs, such as viscoelasticity, hardness, and unique mechanical phenotypes are related to bone disorders, and how they are regulated. Producing a comprehensive view of the ECM of bone disorders is much needed.

## Author contributions

CL: Writing – original draft, Writing – review & editing. XL: Writing – original draft, Writing – review & editing.

